# Unraveling the Impact of Secreted Proteases on Hypervirulence in *Staphylococcus aureus*

**DOI:** 10.1128/mBio.03288-20

**Published:** 2021-02-23

**Authors:** Brittney D. Gimza, Jessica K. Jackson, Andrew M. Frey, Bridget G. Budny, Dale Chaput, Devon N. Rizzo, Lindsey N. Shaw

**Affiliations:** a Department of Cell Biology, Microbiology, and Molecular Biology, University of South Florida, Tampa, Florida, USA; New York University School of Medicine

**Keywords:** *Staphylococcus aureus*, proteases, toxins, virulence regulation

## Abstract

Staphylococcus aureus controls the progression of infection through the coordinated production of extracellular proteases, which selectively modulate virulence determinant stability. This is evidenced by our previous finding that a protease-null strain has a hypervirulent phenotype in a murine model of sepsis, resulting from the unchecked accumulation of virulence factors. Here, we dissect the individual roles of these proteases by constructing and assessing the pathogenic potential of a combinatorial protease mutant library. When strains were constructed bearing increasing numbers of secreted proteases, we observed a variable impact on infectious capacity, where some exhibited hypervirulence, while others phenocopied the wild-type. The common thread for hypervirulent strains was that each lacked both aureolysin and staphopain A. Upon assessment, we found that the combined loss of these two enzymes alone was necessary and sufficient to engender hypervirulence. Using proteomics, we identified a number of important secreted factors, including SPIN, LukA, Sbi, SEK, and PSMα4, as well as an uncharacterized chitinase-related protein (SAUSA300_0964), to be overrepresented in both the *aur scpA* and the protease-null mutants. When assessing the virulence of *aur scpA* SAUSA300_0964 and *aur scpA lukA* mutants, we found that hypervirulence was completely eliminated, whereas *aur scpA spn* and *aur scpA sek* strains elicited aggressive infections akin to the protease double mutant. Collectively, our findings shed light on the influence of extracellular proteases in controlling the infectious process and identifies SAUSA300_0964 as an important new component of the S. aureus virulence factor arsenal.

## INTRODUCTION

Staphylococcus aureus is a Gram-positive pathogen capable of causing an array of serious infections (1). Contributing to the pathogenic complexity of S. aureus is the coordinated production of a collection of virulence factors, including toxins, proteases, hemolysins, nucleases, adhesins, and lipases, which allow the bacteria to evade the immune system, adhere to and colonize host tissues, and acquire nutrients (2–4). With regard to proteases, S. aureus produces 10 major extracellular enzymes that are transcribed from four different loci within the chromosome (5, 6). These include a metalloprotease (*aur*, aureolysin), a serine protease (*sspA*, V8), two cysteine proteases (*scpA* [staphopain A] and *sspB* [staphopain B]), and six serine-like proteases (*splABCDEF*) (6, 7).

In the context of secreted proteases and virulence, several contradictory studies have been performed to determine their function during S. aureus infection. For example, an *sspA* mutant was used in three different animal models of infection and displayed attenuation of virulence in each case (5). In addition, attenuation in virulence was observed for mutations in *sspABC* and *sspBC* in a murine skin abscess model (6). In contrast, an *sspA* mutant displayed increased virulence using a tissue abscess model (8). Conversely, using a murine model of septic arthritis, mutations for *sspABC*, *sspB*, and *aur*, did not display any virulence defect (9). Similarly, it was shown that deletion of *splABCDEF* resulted in no significant virulence defect using a murine peritonitis infection model (7). Other studies with an *spl* operon mutant, this time using a rabbit model of pneumonia, demonstrated that there was no attenuation in overall virulence for the mutant strain; however, severe infection was only induced in one lung, whereas the wild-type affected both lungs equally (10). In addition, using a guinea pig model of vascular permeability, it was shown that ScpA and SspB promote permeability and, as such, can contribute to septic shock (11). Finally, using a murine skin abscess model, it was shown that mutations in *aur* and *scpAB* resulted in no virulence defect (6).

In order to categorically assess the role of S. aureus secreted proteases in virulence, a study by our group focused on analyzing a strain genetically lacking all 10 enzymes (12). In that study, the most notable observation was that, when assessing the infectious capacity of the protease-null strain in a murine model of sepsis, there was a significant increase in mortality for mice infected with this strain in comparison to the wild-type. This hypervirulence was contrary to a number of our other observations from this study, where secreted proteases were shown to be required for resisting phagocytosis, surviving in whole human blood, growing in the presence of antimicrobial peptides, as well as for growth in peptide rich media and serum. To explain these conflicting phenotypes, a proteomic analysis was performed revealing that the enhanced virulence observed was mediated by increased abundance of surface and secreted virulence factors in the mutant strain. Thus, it would appear that in the absence of secreted proteases, the stability and abundance of virulence factors is no longer controlled, allowing for their unregulated accumulation, and consequently one observes more aggressive and deadly infections. Similarly, but in contrast, conditions where the protease-null strain demonstrated ablated growth and/or survival compared to the parental strain are likely the result of protease action directly on the host or host substrates. In support of our findings, these observations were also reported in a companion study by Zielinska et al. (13). As such, we suggest that in addition to cleaving host proteins to facilitate host invasion and immune subversion, the secreted proteases of S. aureus also appear to influence disease progression by controlling the stability of self-derived virulence determinants.

To explore further the pathogenesis related findings from our previous work, we characterize here the individual roles of secreted proteases with regard to the observed hypervirulence. To do this, we constructed and assessed a combinatorial protease mutant library that revealed the enhanced killing observed for the complete protease-null strain during sepsis appears to be driven by the absence of only aureolysin and staphopain A. Further, using a proteomics approach, we identified a number of important secreted factors increased in abundance across both *aur scpA* and protease-null mutants, but not in the parent, including SPIN, LukA, Sbi, and PSMα4, as well as uncharacterized proteins, such as SAUSA300_0964. When these factors were separately inactivated in the hypervirulent *aur scpA* mutant, we observed varied effects on virulence. For example, the creation of an *aur scpA spn* (*spn* encodes SPIN) triple mutant had a partial but nonsignificant impact on the enhanced virulence of the *aur scpA* mutant, whereas an *aur scpA sek* mutant retained a clear, hypervirulent phenotype. Of more interest, we noted that when either SAUSA300_0964 or LukA were separately inactivated in the *aur scpA* mutant, we were able to completely reverse the hypervirulence observed for the double protease mutant. Thus, our study provides unique insight into the role of secreted proteases and the control of S. aureus infection and identifies an entirely new and uncharacterized factor (SAUSA300_0964) as being a major weapon in the arsenal of virulence factors produced by this organism.

## RESULTS

### Generating a combinatorial library of protease mutant strains in *S. aureus*.

**FIG 1:**
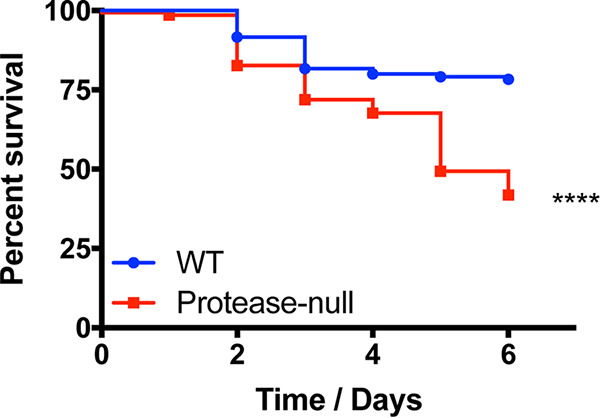
The ablation of S. aureus secreted protease activity results in a hypervirulent phenotype during sepsis. The wild-type (WT) and protease-null mutant were separately inoculated via tail vein injections into groups of 10 CD-1 mice at 1 × 10^8^ cells. Infections were allowed to progress for 6 days or until mice reached a premoribund state (a measure of mortality). Statistical significance was determined using a log rank test (****, *P* < 0.0001 [relative to the wild-type strain]). *n* = 120 mice per strain.

We have previously demonstrated that, upon deletion of all 10 extracellular proteases of S. aureus, a hypervirulent phenotype is observed in a murine model of sepsis (12) (Fig. 1). This finding is explained by a profound increase in virulence factor abundance in the complete protease-null strain (12). Thus, it would appear that the S. aureus secreted proteases exist to modulate the stability of secreted virulence factors so as to regulate infection severity. To explore these findings more fully, we first wanted to establish whether it is necessary to delete all secreted proteases to produce this phenomenon or whether the ablation of only a select few elicited the same outcome. Accordingly, we generated a library of protease mutant strains encompassing every possible combination of disruption (Table 1). It is worth noting that, in these studies, SplABCDEF was considered as a single unit rather than as individual enzymes. This was first a result of simplicity, to reduce the number of strains generated, but also because the Spl enzymes have very narrow substrate specificities (14–16) and likely have only a limited number of individual proteolytic targets. Thus, in these studies, we have five possible protease regions to inactivate: *aur*, *scpA*, *sspA*, *sspB*, and *splABCDEF*.

**TABLE 1:** Strains and plasmids used in this study

Strain or plasmid	Description^*a*^	Reference or source
Strains		
E. coli		
DH5α	Cloning strain	49
S. aureus		
RN4220	Restriction-deficient strain	Lab stock
AH1263	USA300 LAC CA-MRSA; Erm^s^	50
AH1919	USA300 LAC Δ*aur* Δ*sspAB* Δ*scpA spl*::Erm (protease-null)	51
NE163	USA300 JE2 *aur*::Tn::Erm	52
NE1506	USA300 JE2 *sspA*::Tn::Erm	52
NE934	USA300 JE2 *sspB*::Tn::Erm	52
NE1278	USA300 JE2 *scpA*::Tn::Erm	52
KB600	RN6390 *spl*::Erm	7
BDG2485	USA300 LAC *aur*::Tn::Erm	This study
BDG2487	USA300 LAC *sspA*::Tn::Erm	This study
BDG2488	USA300 LAC *sspB*::Tn::Erm	This study
BDG2486	USA300 LAC *scpA*::Tn::Erm	This study
DM2288	USA300 LAC *spl*::Erm	This study
DM2294	USA300 LAC *aur*::Tn::Spc	This study
BB2481	USA300 LAC *aur*::Kan	This study
DM2298	USA300 LAC *sspA*::Tn::Spc	This study
BDG2745	USA300 LAC *sspB*::Kan	This study
DM2293	USA300 LAC *sspB*::Tn::Kan	This study
DM2297	USA300 LAC *sspB*::Tn::Tet	This study
DM2296	USA300 LAC *scpA*::Tn::Tet	This study
BDG2748	USA300 LAC *scpA*::Tn::CM	This study
BDG2391	USA300 LAC *aur*::Tn::Spc *sspA*::Tn::Erm *sspB*::Tn::Kan *scpA*::Tn::Tet	This study
BDG2649	USA300 LAC *aur*::Kan *sspA*::Tn::Spc *sspB*::Tn::Tet *spl*::Erm	This study
BDG2648	USA300 LAC *aur*::Kan *sspA*::Tn::Spc *scpA*::Tn::Tet *spl*::Erm	This study
BDG2392	USA300 LAC *aur*::Tn::Spc *sspB*::Tn::Kan *scpA*::Tn::Tet *spl*::Erm	This study
BDG2750	USA300 LAC *sspA*:Tn::Spc *sspB*::Tn::Tet *scpA*::Tn::CM *spl*::Erm	This study
BDG2640	USA300 LAC *aur*::Kan *sspA*::Tn::Erm *sspB*::Tn::Tet	This study
BDG2643	USA300 LAC *aur*::Kan *sspA*::Tn::Erm *scpA*::Tn::Tet	This study
BDG2639	USA300 LAC *aur*::Kan *sspA*::Tn::Spc *spl*::Erm	This study
BDG2642	USA300 LAC *aur*::Kan *sspB*::Tn::Erm *scpA*::Tn::Tet	This study
BDG2390	USA300 LAC *aur*::Tn::Spc *sspB*::Tn::Erm *scpA*::Tn::Tet	This study
BDG2641	USA300 LAC *aur*::Kan *sspB*::Tn::Tet *spl*::Erm	This study
BDG2644	USA300 LAC *aur*::Kan *scpA*::Tn::Tet *spl*::Erm	This study
BDG2388	USA300 LAC *sspA*::Tn::Spc *sspB*::Tn::Tet *spl*::Erm	This study
BDG2344	USA300 LAC *sspA*::Tn::Spc *scpA*::Tn::Tet *spl*::Erm	This study
BDG2749	USA300 LAC *sspA*::Tn::Spc *sspB*::Tn::Tet *scpA*::Tn::CM	This study
BDG2747	USA300 LAC *sspB*::Kan *scpA*::Tn::Tet *spl*::Erm	This study
BDG2650	USA300 LAC *aur*::Tn::Erm *scpA*::Tn::Tet	This study
BDG2653	USA300 LAC *aur*::Tn::CM *scpA*::Tn::Tet	This study
BDG2758	USA300 LAC *aur*::Kan *scpA*::Tn::Tet	This study
BDG2638	USA300 LAC *aur*::Kan *sspA*::Tn::Spc	This study
BDG2757	USA300 LAC *aur*::Kan *sspB*::Tn:Tet	This study
BDG2389	USA300 LAC *aur*::Tn::Spc *sspB*::Tn::Kan	This study
BDG2489	USA300 LAC *aur*::Tn::Spc *spl*::Erm	This study
BDG2645	USA300 LAC *sspA*::Tn::Spc *sspB*::Tn::Tet	This study
DM2292	USA300 LAC *sspA*::Tn::Erm *scpA*::Tn::Tet	This study
BDG2346	USA300 LAC *sspA*::Tn::Spc *spl*::Erm	This study
BDG2746	USA300 LAC *sspB*::Kan *scpA*::Tn::Tet	This study
DM2291	USA300 LAC *sspB*::Tn::Tet *spl*::Erm	This study
DM2289	USA300 LAC *scpA*::Tn::Tet *spl*::Erm	This study
NE1255	USA300 JE2 *sek*::Tn::Erm	52
NE1300	USA300 JE2 *lukA*::Tn::Erm	52
NE1406	USA300 JE2 *spn*::Tn::Erm	52
BDG2824	USA300 LAC SAUSA300_0964::Kan	This study
BDG2744	USA300 LAC *aur*::Tn::CM *scpA*::Tn::Tet lukA::Tn::Erm	This study
BDG2817	USA300 LAC *aur*::Tn::CM *scpA*::Tn::Tet spn::Tn::Erm	This study
BDG2823	USA300 LAC *aur*::Tn::CM *scpA*::Tn::Tet SAUSA300_0964::Kan	This study
BDG2791	USA300 LAC *aur*::Tn::CM *scpA*::Tn::Tet sek::Tn::Erm	This study
		
Plasmids		
pJB68	Plasmid to create mutants in S. aureus	46
pBB01	pJB38 derived construct for *aur* mutation; Amp^r^ CM^r^	This study
pBDG03	pJB38 derived construct for *sspB* mutation; Amp^r^ CM^r^	This study
pBDG04	Plasmid for chloramphenicol cassette switch (pCM)	This study
pBDG05	pJB68 derived construct for SAUSA300_0964 mutation; Amp^r^ CM^r^ Kan^r^	This study
pKan	Plasmid for kanamycin cassette switch	46
pTet	Plasmid for tetracycline cassette switch	46
pSpc	Plasmid for spectinomycin cassette switch	46

a Resistance cassettes: Erm, erythromycin; Tet, tetracycline; Kan, kanamycin; CM, chloramphenicol; Spc, spectinomycin; Amp, ampicillin.

### The activity of either aureolysin or staphopain A alone limits hypervirulence.

**FIG 2:**
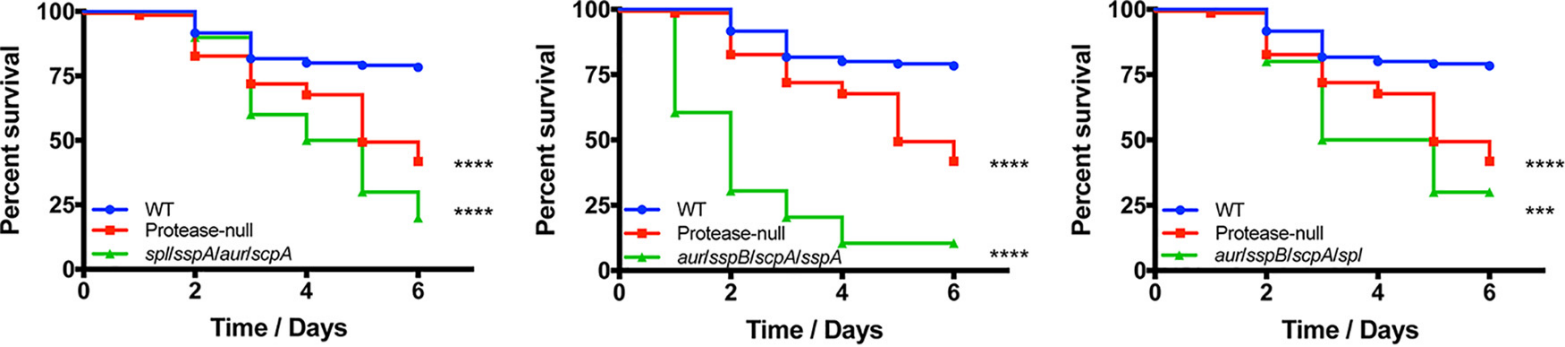
Restoring activity of staphopain B or any of the serine proteases to the protease-null strain does not blunt hypervirulence. The wild-type (WT) and protease mutants noted above were separately inoculated via tail vein injections into groups of 10 CD-1 mice at 1 × 10^8^ cells. Infections were allowed to progress for 6 days or until mice reached a premoribund state (measure of mortality). Statistical significance was determined using a log rank test (***, *P* < 0.001; ****, *P* < 0.0001 [relative to the wild-type strain]). WT and protease-null mutant, *n* = 120 mice per strain; quadruple mutants, *n* = 10 mice per strain.

**FIG 3:**
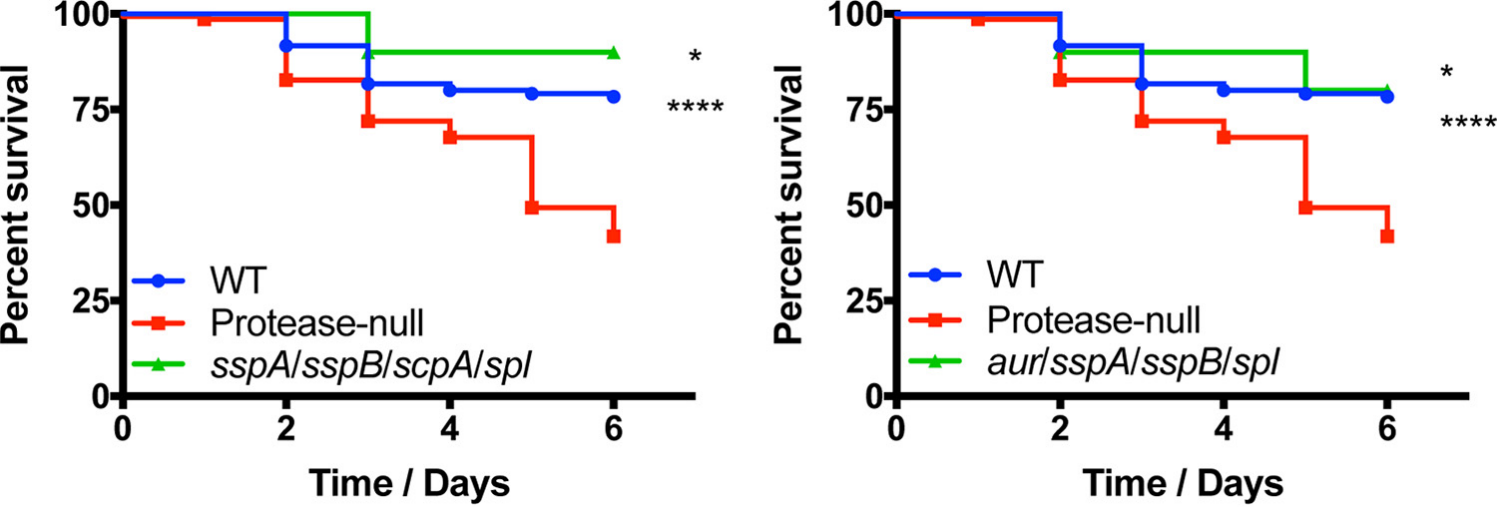
Restoring activity of aureolysin or staphopain A to the protease-null mutant limits hypervirulence during sepsis. The wild-type (WT) and protease mutants noted above were separately inoculated via tail vein injections into groups of 10 CD-1 mice at 1 × 10^8^ cells. Infections were allowed to progress for 6 days or until mice reached a premoribund state (measure of mortality). Statistical significance was determined using a log rank test (*, *P* < 0.05; ****, *P* < 0.0001 [relative to the protease-null mutant]). WT and protease-null mutant, *n* = 120 mice per strain; quadruple mutants, *n* = 10 mice per strain.

Using a murine model of sepsis, we began our analyses by infecting mice with strains lacking all but one of the secreted proteases (with the Spl caveat noted above, resulting in five new strains, see Table 1), alongside the wild-type and protease-null strains. Infections were allowed to progress for 6 days or until mice reached a premoribund state (as a measure of mortality). When reviewing these data, we observed that three of these five mutants displayed enhanced virulence compared to the wild type. Specifically, when comparing these three strains to the protease-null mutant, we noted that the *spl sspA aur scpA* and *aur sspB scpA spl* mutants mirrored the protease-null mutant in its lethality, whereas the *aur sspB scpA sspA* mutant exhibited an even more pronounced hypervirulent phenotype (Fig. 2). The finding with the *aur sspB scpA sspA* mutant is most intriguing and emphasizes the individual roles of the proteases present in each strain for disease progression. Indeed, while there is still clearly an increase in abundance of virulence factors with these mutants (due to the enhanced pathogenic potential), the presence of individual proteases (in this case, the Spl enzymes) appears to aid in the infectious process, resulting in an even more severe infection. This is likely due to the well-documented role of individual S. aureus secreted proteases in facilitating tissue destruction, nutrient generation, and immune evasion during infection (17–20). In contrast to these hypervirulent strains, the other two mutants, *sspA sspB scpA spl* and *aur sspA sspB spl*, displayed wild-type virulence (Fig. 3). Although not definitive, the common theme from these data is that hypervirulence occurs in strains missing both aureolysin (*aur*) and staphopain A (*scpA*), whereas the activity of one or the other of these proteases alone in S. aureus engenders wild-type levels of virulence.

### Only triple mutants lacking both aureolysin and staphopain A together are hypervirulent.

**FIG 4:**
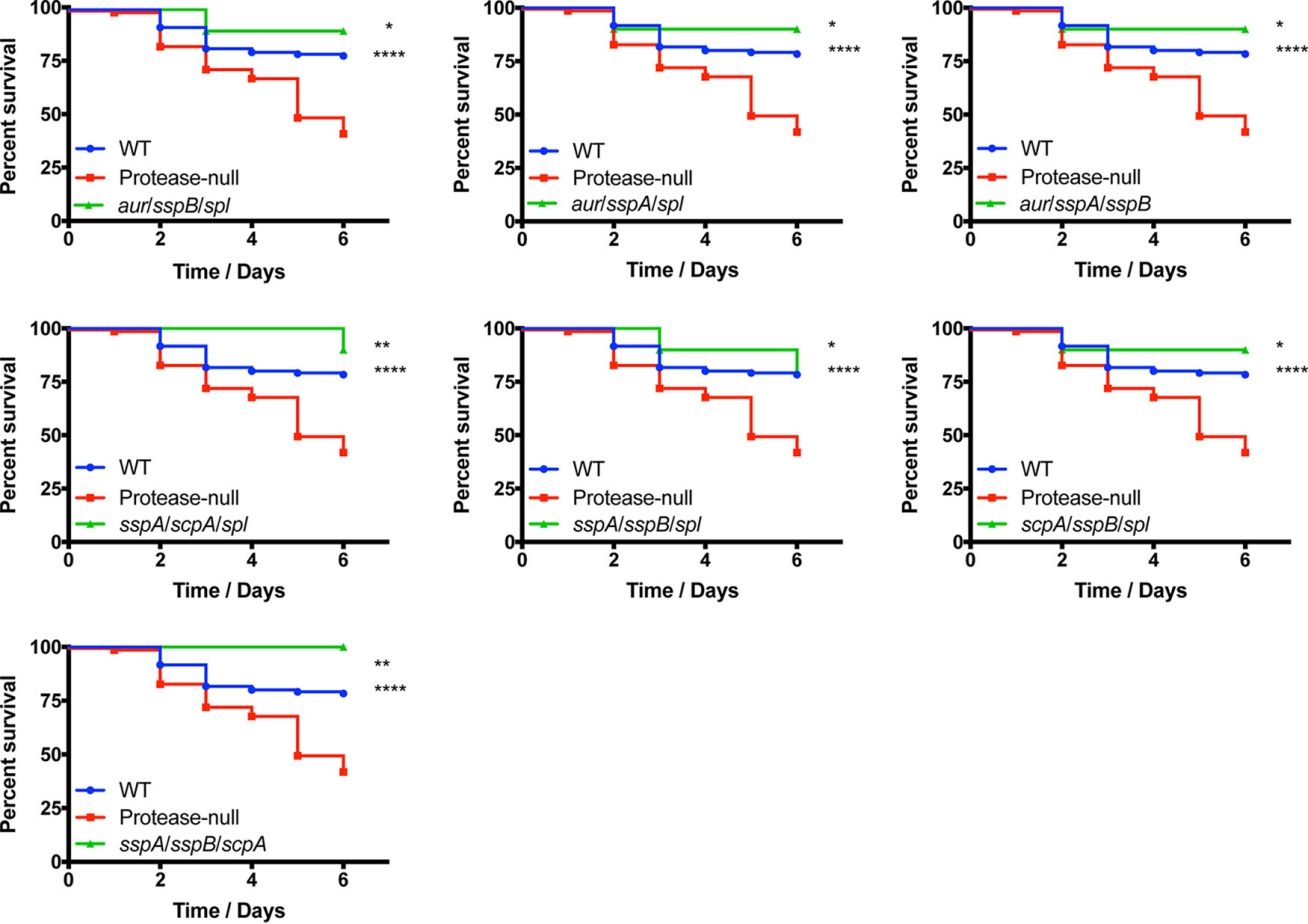
The majority of triple protease mutants do not exhibit a hypervirulent phenotype during sepsis. The wild-type (WT) and protease mutants noted above were separately inoculated via tail vein injections into groups of 10 CD-1 mice at 1 × 10^8^ cells. Infections were allowed to progress for 6 days or until mice reached a premoribund state (measure of mortality). Statistical significance was determined using a log rank test (*, *P* < 0.05; **, *P* < 0.01; ****, *P* < 0.0001 [relative to the protease-null mutant]). WT and protease-null mutant, *n* = 120 mice per strain; triple protease mutants, *n* = 10 mice per strain.

**FIG 5:**
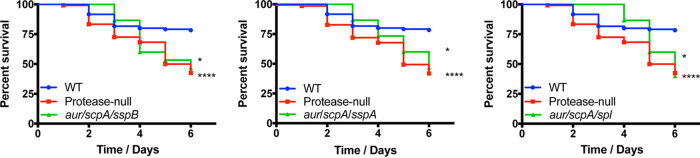
Only triple protease mutants lacking both aureolysin and staphopain A exhibit a hypervirulent phenotype. The wild-type (WT) and protease mutants noted above were separately inoculated via tail vein injections into groups of CD-1 mice at 1 × 10^8^ cells. Infections were allowed to progress for 6 days or until mice reached a premoribund state (measure of mortality). Statistical significance was determined using a log rank test (*, *P* < 0.05; ****, *P* < 0.0001 [relative to the wild-type strain]). WT and protease-null mutant, *n* = 120 mice per strain; triple mutants, *n* = 15 mice per strain.

To continue exploring the importance of the specific proteases to the hypervirulent phenotype, we next assessed the pathogenic potential of strains containing combinations of two different proteolytic enzymes. Accordingly, the 10 different combinations of triple protease deletion mutants were tested, alongside the wild-type and protease-null strain, in the murine model of sepsis. Interestingly, with these mutants we observed a mixture of wild-type and hypervirulent phenotypes. For seven of the combinatorial protease mutants (*aur sspB spl*, *aur sspA spl*, *aur sspA sspB*, *sspA scpA spl*, *sspA sspB spl*, *scpA sspB spl*, and *sspA sspB scpA*), we observed mortality rates that were equal to, or even less pronounced than, that of the wild-type strain (Fig. 4). In contrast, we found that the *aur scpA sspA*, *aur scpA sspB*, and *aur scpA spl* mutants all elicited mortality at levels as severe as the protease-null strain (Fig. 5). When analyzing the triple mutant data more closely, it became apparent that the common thread for a hypervirulent phenotype was that all strains displaying this trait lacked both aureolysin (*aur*) and staphopain A (*scpA*) and that the other enzyme missing alongside these two did not result in an observable phenotype in the murine model of sepsis. This is further supported by our quadruple mutant data where the select mutants exhibiting the hypervirulent phenotype are also lacking both aureolysin and staphopain A, and the activity of either enzyme alone results in the elimination of this phenotype.

### The combined loss of aureolysin and staphopain A activity is necessary and sufficient for hypervirulence in *S. aureus*.

**FIG 6:**
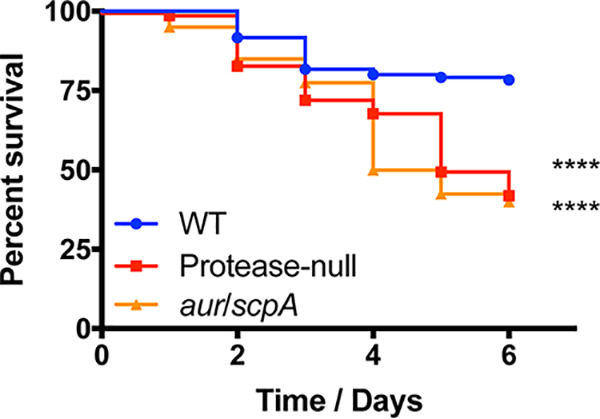
The combined loss of aureolysin and staphopain A is necessary and sufficient to generate hypervirulence in S. aureus. The wild-type (WT), protease-null mutant, and *aur scpA* mutant were separately inoculated via tail vein injections into groups of 10 CD-1 mice at 1 × 10^8^ cells. Infections were allowed to progress for 6 days or until mice reached a premoribund state (measure of mortality). Statistical significance was determined using a log rank test (****, *P* < 0.0001 [relative to the wild-type strain]). WT and protease-null mutant, *n* = 120 mice per strain; *aur scpA* mutant, *n* = 40 mice.

To determine whether in fact the combined loss of *aur* and *scpA* alone was sufficient to generate a hypervirulent phenotype, we next assessed the pathogenic potential of an *aur scpA* mutant, alongside our wild-type and protease-null mutant strains. In so doing, we determined that the pathogenic potential of an *aur scpA* mutant mirrored that of the protease-null strain, not the wild-type (Fig. 6). Thus, it is clear that these two combined mutations together are the minimum requirement for generating a hypervirulent phenotype in S. aureus. Although the virulence phenotypes of single *aur* and *scpA* mutants have been reported numerous times, with no hypervirulence observed, for completeness we also tested these individual strains in our model, confirming that neither has enhanced virulence (see Fig. S2 in the supplemental material). As such, this suggests that together aureolysin and staphopain A modulate the stability of virulence factors in a manner that limits the severity of infection under standard conditions, with profound impacts on disease progression.

### Identification of virulence factors that may contribute to the hypervirulent phenotypes of the protease-null and *aur scpA* mutant strains.

**FIG 7:**
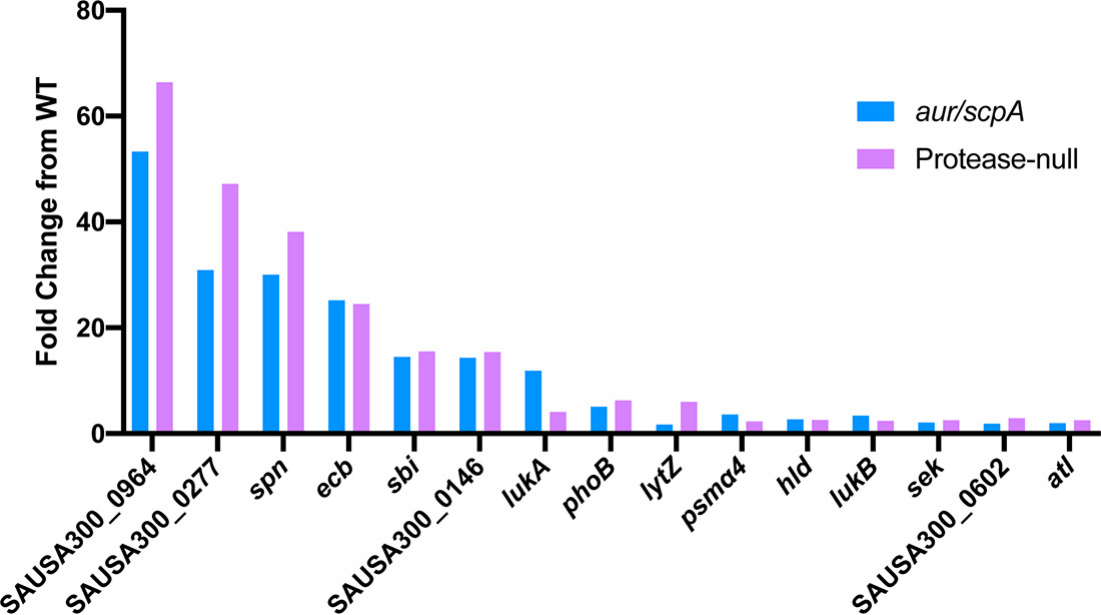
Increased abundance for a wealth of known S. aureus virulence factors is observed in hypervirulent protease mutant strains. Secretomes were isolated from 15 h cultures of wild-type, *aur scpA* mutant, and protease-null mutant strains. Proteins were separated using SDS-PAGE and subjected to mass spectrometric analysis. Full-length proteins were identified at each mass fraction, and the fold change in LFQ intensity was determined for the *aur scpA* and protease-null mutants in comparison to the wild-type.

As proposed previously (12), in the absence of secreted proteases, S. aureus virulence factors exist unchecked, leading to their enhanced accumulation and, ultimately, a hypervirulent phenotype. Thus, having identified the loss of aureolysin and staphopain A activity as being necessary and sufficient for hypervirulence, we next wanted to narrow in on the virulence factor substrates of these two enzymes and thus potentially identify those proving causative for this phenotype. To do this, we determined which secreted factors had increased abundance in both protease-null and *aur scpA* mutant strains but not in the wild-type. As such, secretomes were harvested from wild-type, protease-null, and *aur scpA* mutant strains after 15 h growth, which were then separated by SDS-PAGE. Next, each sample lane of the gel was sectioned into 10 fragments that were subjected to mass spectrometric analysis. These data were processed as the fold change from the wild-type across each mass fraction (see Table S2 in the supplemental material). When setting the threshold for significance to ≥1.5-fold, we identified 15 secreted proteins that showed increased abundance across the *aur scpA* and protease-null mutants compared to the wild-type strain (Fig. 7). Of these changes, the greatest difference was for SAUSA300_0964, which has been designated a chitinase-related protein. Specifically, it demonstrated a 53.3-fold increase in abundance for the *aur scpA* mutant and a 66.4-fold increase for the protease-null strain. The second greatest difference observed was for SAUSA300_0277, which encodes the peptidoglycan hydrolase EssH (21). In the *aur scpA* mutant a 30.9-fold increase in abundance was observed, whereas the protease-null mutant demonstrated a 47.2-fold increase. With regard to better known virulence factors, we observed increases in abundance for: the staphylococcal peroxidase inhibitor SPIN (*aur scpA*, 30-fold; protease-null, 38.1-fold), immunoglobulin binding protein Sbi (*aur scpA*, 14.5-fold; protease null, 15.5-fold), LukA leukotoxin (*aur scpA*, 11.9-fold; protease null, 4.1-fold), phenol-soluble modulin alpha 4 (*aur scpA*, 3.6-fold; protease null, 2.3-fold), delta hemolysin (*aur scpA*, 2.7-fold; protease null, 2.6-fold), LukB leukotoxin (*aur scpA*, 3.4-fold; protease null, 2.4-fold), enterotoxin K (*aur scpA*, 2.1-fold; protease null, 2.5-fold), and the major autolysin (*aur scpA*, 2-fold; protease null, 2.5-fold). Given that, other than SAUSA300_0964, each of these are well-studied virulence factors that are known to play key roles in the S. aureus infectious process; they represent an excellent list of candidates for mediating the hypervirulent phenotypes observed for our protease mutant strains.

### The inactivation of individual virulence factors eliminates the development of hypervirulence in an *aur scpA* mutant.

**FIG 8:**
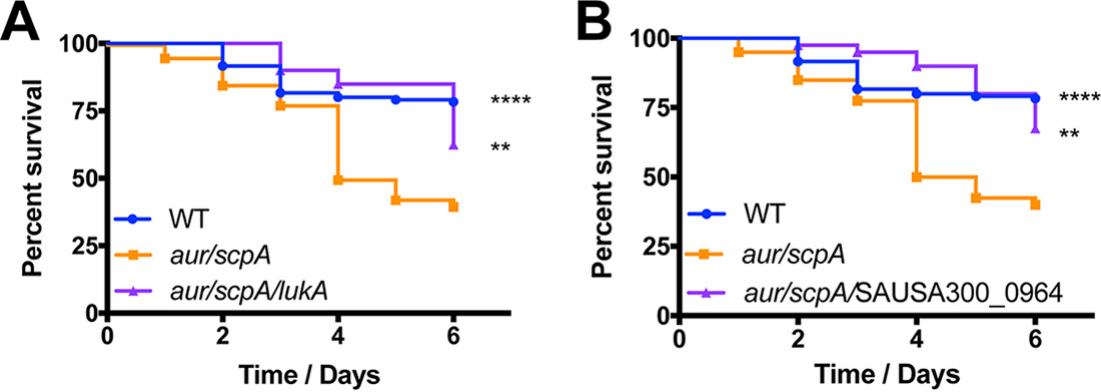
Disruption of SAUSA300_0964 or *lukA* eliminates the hypervirulence of the *aur scpA* mutant. Wild-type (WT), *aur scpA* mutant, and either *aur scpA* SAUSA300_0964 (A) or *aur scpA lukA-*mutant (B) strains were separately inoculated via tail vein injections into groups of 10 CD-1 mice at 1 × 10^8^ cells. Infections were allowed to progress for 6 days or until mice reached a premoribund state (measure of mortality). Statistical significance was determined using a log rank test (**, *P* < 0.01; ****, *P* < 0.0001 [all relative to the *aur scpA* mutant]). WT, *n* = 120 mice per strain; *aur scpA*, *aur scpA* SAUSA300_0964, and *aur scpA lukA*, *n* = 40 mice per strain.

To determine the importance of hyperabundant virulence factors in the *aur scpA* and protease-null mutant strains, we next set out to assess their impact during infection. We began with SAUSA300_0964, an uncharacterized chitinase-related protein, since it had the most substantial increase in abundance (>53-fold) for any secreted protein in the *aur scpA* mutant. Accordingly, mice were separately infected with the wild-type and either the *aur scpA* mutant or an *aur scpA* SAUSA300_0964 mutant. Strikingly, we observed a complete reversal of the *aur scpA* hypervirulent phenotype when SAUSA300_0964 was also inactivated in this strain, displaying a phenotype that appeared to be less virulent than our wild-type strain, with limited mortality on days 2 to 4 of the infectious period (Fig. 8A). Thus, SAUSA300_0964 appears to be a novel virulence factor that is a major contributor to the hypervirulence of S. aureus engendered by protease deletion.

We next assessed the importance of LukA (11.9-fold increase in the double mutant), a well-characterized pore-forming leukotoxin that has been previously shown to contribute to increased virulence in S. aureus strains (22). Upon analysis, we again observed a reduction in hypervirulence when an *aur scpA lukA* strain was compared to its hypervirulent *aur scpA* parent (Fig. 8B). For this strain, we observed limited mortality over the first 5 days of infection that was at a less-pronounced rate than even the wild-type. We did, however, note an upward trend in death toward the end of the infectious period, but at a level similar to the wild-type, and far less severe than the hypervirulent *aur scpA* mutant. As such, these data indicate that LukA also significantly contributes to the aggressive virulence phenotype observed for our protease mutant strains.

**FIG 9:**
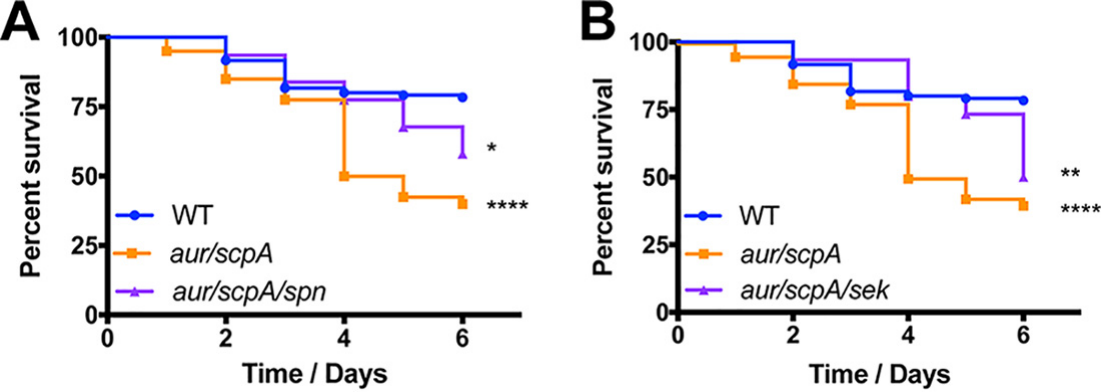
The disruption of *spn* or *sek* in the *aur scpA* mutant does not significantly limit hypervirulence during sepsis. Wild-type (WT), *aur scpA* mutant, and either *aur scpA spn* mutant (A) or *aur scpA sek* mutant (B) strains were separately inoculated via tail vein injections into groups of 10 CD-1 mice at 1 × 10^8^ cells. Infections were allowed to progress for 6 days or until mice reached a premoribund state (measure of mortality). Statistical significance was determined using a log rank test (*, *P* < 0.05; **, *P* < 0.01; ****, *P* < 0.0001; relative to the wild-type strain). WT, *n* = 120 mice per strain; *aur scpA*, *aur scpA spn*, and *aur scpA sek*, *n* = 40 mice per strain.

To determine whether other virulence factors were also able to limit or eliminate the hypervirulent phenotype of our protease mutant strains, we next considered other virulence factors found to be hyperabundant in these strains. We next chose to assess the importance of SPIN, which had a 30-fold increase in stability in the *aur scpA* mutant strain. SPIN is a myeloperoxidase inhibitor that has previously been shown to contribute to immune evasion by hindering MPO-mediated killing (23). Upon analysis, we observed that an *aur scpA spn* mutant displayed an intermediate hypervirulence phenotype between its parent and wild-type strains (Fig. 9A). Interestingly, the *aur scpA spn* mutant closely mirrored wild-type virulence until day 5, at which point survival rates dropped to near that of the *aur scpA* mutant strain. As such, these data suggest that SPIN perhaps contributes, but is not by itself sufficient, for hypervirulence resulting from protease deletion.

Lastly, we evaluated the role of SEK, an enterotoxin (a 2.1-fold increase in the double protease mutant) that has been previously shown to contribute to virulence in murine models of sepsis (24). Here, we noted that, when *sek* was disrupted in the double protease mutant, we observed an initial lag in mortality between days 2 and 5 of the infection, at which point the percent survival of mice plummeted to a level akin to the *aur scpA* mutant (Fig. 9B). As such, it would appear that although SEK may contribute in some minor way to hypervirulence upon protease disruption, its impact is perhaps minimal at best.

## DISCUSSION

The pathogenic success of S. aureus is largely attributed to the repertoire of virulence factors it produces in the human host. With regard to secreted proteases, their importance for disease causation has only until recently been clarified by a study from our group, where we reveal that these enzymes modulate the severity of infection by controlling virulence factor stability and abundance (12). This is evidenced by our finding that a protease-null strain has a substantially increased infectious capacity in a murine model of sepsis, resulting from the unchecked accumulation of virulence determinants. A limitation to our previous study, however, is that we only assessed the pathogenic potential of a strain genetically lacking all 10 secreted proteases. As such, in this study we followed up on these earlier findings by assessing the individual importance of S. aureus secreted proteases by constructing and assessing a combinatorial protease mutant library using the same murine model of infection. Here, we found that S. aureus strains bearing only *sspA*, *sspB*, or the *spl* proteases alone still displayed enhanced virulence, akin to that previously demonstrated by the protease-null mutant. Conversely, strains that possessed either aureolysin (Aur) or staphopain A (ScpA) activity displayed wild-type virulence, suggesting a role for these proteases in the hypervirulent phenotype. This is perhaps logical, since previous studies have shown that aureolysin can specifically cleave alpha-hemolysin (Hla) (25) and the phenol-soluble modulins (PSMs) (26), whereas staphopain A has been shown to control the abundance of biofilm components (27) and contribute to immune evasion by blocking neutrophil recruitment via cleavage of the CXC chemokine receptor (CXCR2) (28). In addition, a strain that lacked all proteases but the Spl enzymes resulted in even more severe infections than the null strain, likely as a result of the influence of these proteases themselves on disease progression. Of note, SplA has been shown to contribute to bacterial invasion and dissemination in a rabbit model of pneumonia through cleavage of mucin 16 (10). As such, these data emphasize that the targeting of host proteins by secreted proteases plays a significant role in the infection process as well.

When assessing strains containing only two proteolytic enzymes (triple mutants), we observed a similarly variable impact on hypervirulence, since the strains exhibited a mixture of virulent and hypervirulent phenotypes. We noted that each of the triple mutant strains demonstrating a hypervirulent phenotype lacked both aureolysin and staphopain A, further supporting our data suggesting that these two proteases substantially contribute to the hypervirulent phenotype. In contrast, all other triple mutant strains (containing either one or both of these enzymes) demonstrated wild-type virulence. Indeed, when assessed together as a minimally deleted unit, we determined that disruption of aureolysin and staphopain A activity alone is sufficient to generate hypervirulence. When one considers this finding in hindsight, it was perhaps a predictable outcome, since aureolysin and staphopain A are known to have the broadest substrate specificity of all S. aureus proteases (29, 30). Indeed, the Spl enzymes are known to be among the most selective of proteases with regard to substrates (14–16, 31, 32), paralleling their close homologs, the exfoliative toxins, which still have only a single known target (desmogelin-1) (33). Moreover, SspA (V8) is so selective in its cleavage preference that it can be used as an alternative to trypsin in mass spectrometry studies due to its enzymatic reliability (34).

A consideration with the finding that loss of *aur* and *scpA* is the minimal set of proteases for hypervirulence is that a number of the secreted proteases of S. aureus are activated as part of a cascade, whereby aureolysin attains activity via autocatalysis (35) and then in turn activates SspA (36, 37), which subsequently processes the zymogen form of staphopain B into an active enzyme (8, 17). For completeness, it should be noted that staphopain A is also produced as a zymogen but does not require the activity of other secreted proteases to become functional (6), whereas the Spl enzymes are secreted as active proteases (e.g., they lack a profragment) (7). Thus, at face value, one might suggest that an *aur scpA* mutant would only possess the activity of the Spl enzymes, since the loss of aureolysin would abrogate SspA and staphopain B activity. This, however, is not the case, since it is well documented in the literature that SspA can aberrantly process itself into a functional form (6). Similarly, it is also known that staphopain B can become proteolytically active, even in the absence of SspA (8). To ensure that such contentions actually manifest themselves in the *aur scpA* mutant, however, we subjected this strain to analysis via zymography. As expected, an abundant proteolytic activity is present in culture supernatants for this strain that exists between the null phenotype of the complete deletion strain and the strongly proteolytic nature of the wild-type (see Fig. S3).

Obviously, it is not the loss of the aureolysin and staphopain A proteins that results in hypervirulence, but more the loss of their action on specific virulence factors, which in turn has a profound impact on disease progression. When we assessed alterations in secreted factor abundance in our various strains, we observed increased stability in the *aur scpA* mutant for PSMα4, suggesting that its cleavage or the lack thereof by aureolysin could influence disease progression. In addition to this, we found several other commonly known factors, such as Ecb, LukAB, SPIN, and Sek, that were increased in abundance. Importantly, these factors are known to play key roles in the infectious process. For example, LukAB specifically targets human neutrophils (38), and strains lacking *lukAB* are significantly decreased in viability during murine renal abscess infections (22). Similarly, it has been shown that mortality is decreased in a murine sepsis model when mice are immunized with SEK-specific monoclonal antibodies (24). In the context of SPIN, it has been shown that its absence leads to decreased survival of S. aureus following phagocytosis by neutrophils (23). Lastly, Ecb has been shown to contribute to immune evasion by preventing the recognition of opsonized bacteria by neutrophils (39). As such, an increase in the abundance of any of these factors could contribute the more severe infections observed here.

Most profoundly, however, was the increase in abundance of a previously uncharacterized secretory protein in the protease-null and *aur scpA* mutants. Here, we noted a 53-fold increase in abundance for the chitinase-related protein SAUSA300_0964 in the *aur scpA* mutant, and a 66-fold increase for the protease-null strain. Chitinases are enzymes that breaks down chitin by hydrolyzing glycosidic bonds and are found in all kingdoms of life (40). With regard to bacteria, while chitinases are important for nutrient acquisition in marine and soil-dwelling bacteria, their role in nonchitinous hosts is not completely understood (41). However, when assessed in pathogens such as Listeria monocytogenes and Legionella pneumophila, chitinases have been found to be important for virulence using murine models of infection (41–43). Interestingly, whereas no studies in S. aureus have specifically focused on the importance of this chitinase-related protein, its abundance has been found as altered in various strains, including mutants of *spl* (10), *saeR* (44), and *xdrA* (our unpublished data). When we assessed the impact of SAUSA300_0964 to the enhanced virulence of the *aur scpA* mutant, we observed a complete collapse of enhanced infection, with the *aur scpA* SAUSA300_0964 mutant perhaps displaying reduced infectious capacity even compared to the wild type. This clearly suggests that this protein plays an important and previously unidentified role in S. aureus virulence. Indeed, dissecting how SAUSA300_0964 contributes to S. aureus pathogenesis is a major and ongoing focus of study in our laboratory.

The individual impact of LukA to the hypervirulence phenotype was also explored here using an *aur scpA lukA* mutant strain. Although the increases in abundance of LukA in our protease-null mutants were not as substantial as with other secreted factors, its possible contribution to the observed hypervirulence phenotype remained of interest given the previously identified role of LukA in S. aureus disease causation. Interestingly, the absence of *lukA* in the *aur scpA* mutant resulted in a significant reduction in the enhanced virulence observed with *aur scpA* mutant, suggesting that LukA plays an important role in the hypervirulence phenotype observed upon protease removal. Given that LukAB specifically target human neutrophils, it is likely that the contribution of LukA to hypervirulence results from receptor-independent immunomodulatory mechanisms which have been shown for Panton-Valentine leukocidin (PVL) (38). Specifically, although PVL also displays specificity toward human polymorphonuclear cells (PMNs), it has been shown to induce proinflammatory responses in murine and human PMNs and macrophages.

Another secreted factor with a substantial increase in abundance in our protease-null mutant strains was the staphylococcal inhibitor of myeloperoxidase, SPIN. Although an *aur scpA spn* mutant ultimately exhibited enhanced virulence in comparison to the wild-type strain, there appeared to be a delay in mortality suggesting that the absence of *spn* in our *aur scpA* mutant slows disease progression. However, since hypervirulence was still observed with *aur scpA spn* mutant, it is clear that SPIN alone contributes to hypervirulence in only a limited way. Similarly, we investigated the importance of SEK in the enhanced virulence phenotype observed upon protease removal. Although the importance of SEK to S. aureus virulence has been shown in previous studies, we observed that the removal of *sek* from our *aur scpA* mutant does not result in a significant reduction in mortality in comparison to the *aur scpA* mutant. Given that the increase in abundance of SEK in the protease-null mutants (*aur scpA*, 2.1-fold; protease null, 2.5-fold) is relatively minor, it is perhaps not surprising that its absence does not result in substantial alterations in the hypervirulence phenotype.

In summary, this study solidifies the importance of secreted proteases for S. aureus virulence and, more specifically, demonstrates the significant importance of aureolysin and staphopain A. Our findings support the role of secreted proteases as mediators of virulence factor abundance and identifies new and existing virulence determinants that significantly contribute to disease progression. Collectively, our novel findings shed light on the influence of extracellular proteases and the manner in which the infectious process is regulated in S. aureus.

## MATERIALS AND METHODS

### Media and growth conditions.

All bacterial strains and plasmids used here are listed in Table 1. All overnight cultures were grown at 37°C with shaking at 250 rpm in 5 ml of either lysogeny broth (LB) or tryptic soy broth (TSB). When required, the following concentrations of antibiotics were added: for Escherichia coli, 100 μg ml^−1^ ampicillin; and for S. aureus, 5 μg ml^−1^ tetracycline, 5 μg ml^−1^ erythromycin, 25 μg ml^−1^ lincomycin, 50 μg ml^−1^ kanamycin, 50 μg ml^−1^ neomycin, 1,000 μg ml^−1^ spectinomycin, and 2.5 μg ml^−1^ chloramphenicol. When experiments required synchronous cultures of S. aureus, overnight cultures were diluted 1:100 into 5 ml of fresh TSB and grown for 3 h, at which point they were standardized to an optical density at 600 nm of 0.05 in 100 ml of TSB in a 250-ml flask.

### Construction of single protease mutants.

Transposon mutants in S. aureus USA300 JE2 for *aur*, *sspA*, *sspB*, and *scpA* were obtained from the Nebraska Transposon Mutant Library (NTML). The *spl* operon mutant in S. aureus RN6390 was previously constructed by Reed et al. (7). Each mutant was transduced into the USA300 LAC wild-type strain using phi11, as described by us previously (45), and confirmed by PCR using gene-specific primers (*aur*, OL535/OL3402; *sspA*, OL3528/OL3529; *sspB*, OL147/OL148; *scpA*, OL15/OL16; *spl*, OL1979/OL3872). All primers used in this study are listed in Table S1 in the supplemental material.

10.1128/mBio.03288-20.4 TABLE S1 Primers used in this study. Restriction sites are underlined lowercase letters. Download Table S1, PDF file, 0.03 MB. Copyright © 2021 Gimza et al. 2021 Gimza et al. https://creativecommons.org/licenses/by/4.0/ This content is distributed under the terms of the Creative Commons Attribution 4.0 International license.

10.1128/mBio.03288-20.5 TABLE S2 Secreted proteins with increased abundance in both the *aur scpA* mutant and protease-null mutant strains but not in the wild type. Download Table S2, PDF file, 0.04 MB. Copyright © 2021 Gimza et al. 2021 Gimza et al. https://creativecommons.org/licenses/by/4.0/ This content is distributed under the terms of the Creative Commons Attribution 4.0 International license.

### Generation of a chloramphenicol allelic replacement vector.

In order to create a combinatorial protease-mutant library, several of the protease mutants needed to be constructed containing different antibiotic resistance cassettes to facilitate selection for multiple chromosomal mutations. Although allelic switch vectors exist, allowing for the replacement of NTML transposons with different antibiotic markers, the complexity of our library required additional options. Accordingly, a new allelic-exchange plasmid, pCM, was constructed in this study (pBG04), allowing us to substitute the transposon with a chloramphenicol resistance cassette. To construct pBG04, an inverse PCR was performed on pTET using OL4473 and OL4474 to amplify the entire plasmid, excluding its tetracycline resistance cassette. A chloramphenicol resistance cassette was amplified from pKOR1 using OL4563 and OL4564. Next, these fragments were ligated together using SacI sites. This was then transformed into chemically competent E. coli, with clones confirmed by PCR using gene-specific and plasmid-specific primers OL4618 and OL4619.

### Allelic-replacement strategies for changing antibiotic resistance markers within protease mutant strains.

To create protease mutants with altered antibiotics resistance markers (*aur*::Tn::*cm*, *aur*::Tn::*spc*, *sspA*::Tn::*spc*, *sspB*::Tn::*kan*, *sspB*::Tn::*tet*, *scpA*::Tn::*tet*, *scpA*::Tn::*cm*), allelic-exchange strategies were used. The previously described allelic-exchange plasmids pTet, pKan, and pSpc were used alongside the newly created pCM to replace the transposon in mutants with a tetracycline, kanamycin, spectinomycin, or chloramphenicol resistance cassette, respectively (46). All resistance cassette switches were transduced into a clean USA300 LAC wild-type background and confirmed by PCR using the same gene-specific primers noted above, apart from *sspB*::Tn::*tet* (OL148/OL3496) and *scpA*::Tn::*tet* (OL15/OL3496), where gene- and tetracycline cassette-specific primers were used due to the similarity in the sizes of the erythromycin and tetracycline resistance cassettes.

### Generation of deletion mutants for aureolysin, staphopain B, and SAUSA300_0964.

Despite these alterations, we still found it necessary to create a *de novo* deletion mutant for the *aur* gene. A kanamycin marked deletion was thus generated using plasmid pJB38, as described by Bose et al. (46). Using the primer pairs OL4212/OL4213 and OL4214/OL4215, two fragments containing the regions up and downstream of *aur*, along with small portions of the 5′ and 3′ ends of the gene, were amplified by PCR. A kanamycin resistance cassette was amplified using OL4220 and OL4221 from a 8325-4 *sarA*::*kan* mutant (47). The kanamycin cassette was ligated between the two previously generated fragments using a MluI site, and then the ligated fragment was cloned into pJB38 using EcoRI and KpnI sites. Deletion of the majority of the *aur* gene was then performed using an established protocol of allelic replacement (46). To confirm correct construction, the region where the deletion occurred was amplified by PCR using OL4212 and OL4215 and subjected to sequencing. Similarly, a new kanamycin-marked *sspB* deletion was also constructed here according to the method described above. The flanking fragments were generated using OL4216/OL4217 and OL4218/OL4219. The kanamycin cassette was ligated in between the two fragments using a BglII site. This fragment was then cloned into pJB38 using XhoI and KpnI sites. The *sspB* deletion was confirmed using the primers OL4216 and OL4219. A SAUSA300_0964 deletion mutant was constructed because no transposon mutant is contained within the NTML library. Using the method described above, a kanamycin marked SAUSA300_0964 deletion was constructed. The fragments surrounding the gene were generated using OL5125/OL5126 and OL5127/OL5128. The kanamycin cassette was ligated in between the two fragments using a BglII site. The fragment was then cloned into pJB38 using EcoRI and KpnI. The SAUSA300_0964 deletion was confirmed using the primers OL5125 and OL5128.

### Construction of protease and virulence factor triple mutants.

To construct the *aur scpA lukA*, *aur scpA spn*, and *aur scpA sek* triple mutants, the *lukA*, *spn*, and *sek* transposon mutants were obtained from the NTML library. Separately, the *lukA*, *spn*, and *sek* mutations were transduced into the *aur*::Tn::CM *scpA*::Tn::Tet mutant, using phi11 as described above, and confirmed by PCR using gene-specific primers (*lukA*, OL5135/OL5136; *spn*, OL5679/OL5680; and *sek*, OL5315/OL5316). The *aur scpA* SAUSA300_0964 mutant was similarly created by transducing the *aur*::Tn::CM *scpA*::Tn::Tet mutant with the SAUSA300_0964 mutation, and confirmed using the primers detailed above.

### Murine model of bacterial sepsis.

All animal studies in this work were performed in accordance with and approved by the Institutional Animal Care and Use Committee of the University of South Florida (permit A-4100-01). These experiments were carried out as previously described (12). To prepare cultures for infection, aliquots of overnight cultures were washed twice and diluted to 1 × 10^9^ CFU/ml in phosphate-buffered saline (PBS). Next, 6-week-old CD-1 female mice purchased from Charles River Laboratories were separately inoculated by tail vein injection with 100 μl of culture (final inoculum, 1 × 10^8^ CFU/ml). The infection was then allowed to progress for 6 days or until mice reached a premoribund state (used as a measure of mortality), at which point they were euthanized. The criteria for a premoribund state in mice was defined as follows: hunched posture, rapid and/or labored breathing, ruffled fur, lethargy, failure to respond to stimuli, soiled anogenital area, paralysis, head tilt, circling, vocalizations, nonpurposeful movements, and/or are unable to eat or drink. Final survival graphs were generated by pooling wild-type, protease-null, and *aur scpA* mutant data from individual experiments. The percent survival of mice infected with our various strains was determined and compared using a log rank test to determine statistical significance.

### Proteomic analysis of secretomes.

Secreted proteins were isolated from stationary-phase (15 h) cultures of wild-type, protease-null mutant, and the *aur scpA* mutant strains. To do this, cultures were centrifuged, and the supernatants were placed in a fresh tube, prior to the addition of 10% trichloroacetic acid for protein precipitation. Samples were incubated overnight at 4°C and then centrifuged to pellet the precipitated protein. These precipitates were washed with 100% ethanol three times, resuspended in PBS, and quantified by using a Pierce 660-nm kit (Thermo Fisher Scientific). For size selection, 25 μg of each protein sample was denatured at 95°C for 5 min in Laemmli buffer, subjected to electrophoresis on a 12% SDS-PAGE gel, and visualized using Instant Blue. After this, each sample lane of the gel was cut into 10 pieces with specific mass ranges determined using the protein ladder (depicted in Fig. S1). Each gel piece was then placed into individual microcentrifuge tubes. To remove the SDS and Instant Blue stain, the gel pieces were washed twice by adding 200 μl of 50:50 acetonitrile (ACN)-ammonium bicarbonate (ABC) to the sample tubes, followed by vortexing for 15 min and then removal of the wash. To dehydrate gel pieces, ACN was added to samples, followed by incubation for 5 to 10 min or until the gel pieces turned white. Next, gel pieces were resuspended in 20 to 50 μl (determined by the size of the gel piece) of 100 mM ABC and incubated for 5 min to rehydrate gel pieces. To achieve a 1:1 ratio of CAN to 100 mM ABC, an equal volume of ACN was added, and the samples were then vortexed for 15 min. Next, the excess wash was removed, and the samples were placed in a SpeedVac for 5 min to dry. For gel digestion, 100 μl of 50 mM dithiothreitol (DTT) was added to samples for rehydration of gel pieces. Samples were then incubated at 55°C for 30 min before cooling to room temperature. The DTT was removed via pipetting, 100 μl of 100 mM iodoacetamide was added, and the samples were incubated in the dark for 30 min. Next, the buffer was removed by pipetting, and the fragments were washed and vortexed for 15 min in 100 μl of 50:50 ACN–100 mM ABC. To dry samples, the buffer was removed, and the samples were placed in a SpeedVac for 5 min. Samples were then placed on ice for 5 min before the addition of cold trypsin and incubation for 30 min. The samples were then placed at 37°C and incubated overnight. The next day, supernatants containing peptides were transferred to fresh microcentrifuge tubes. To further extract peptides from gel pieces, the samples were washed twice by adding 200 μl of 50:50 CAN–water in 0.1% formic acid (FA) with vortexing for 15 min. Each time, the wash was added to the tube containing the supernatant. The samples were then placed in a SpeedVac until completely dry, before being resuspended in 20 μl of 0.1% FA and placed into an ultrasonic water bath for 10 min. Then, the samples were centrifuged at 15,000 × *g* for 10 min with the supernatant subsequently transferred into autosampler vials. Aliquots (5 μl) were separated on a 50-cm Acclaim PepMap 100 C_18_ reversed-phase high-pressure liquid chromatography (HPLC) column (Thermo Fisher Scientific) using an Ultimate3000 UHPLC (Thermo Fisher Scientific), with a 120-min gradient (2 to 32% ACN with 0.1% FA). Peptides were analyzed on a hybrid Quadrupole-Orbitrap instrument (Q Exactive Plus; Thermo Fisher Scientific) using data-dependent acquisition in which the top 10 most abundant ions were selected for tandem mass spectrometry analysis. Each sample was analyzed as separate fractions to identify intact proteins (i.e., of their predicted mass). Proteins were included for further analysis only if their predicted molecular weight (in kDa) fell within that of their mass fraction (as observed on the SDS-PAGE gel, and proteins with a predicted mass within ±20% of the upper size threshold of the fraction were considered “intact” and included for analysis). The LFQ (label-free quantification) intensity was used to establish fold change in intact proteins between WT and mutants.

10.1128/mBio.03288-20.1 FIG S1 Depiction of SDS-PAGE gel sectioning by mass fraction for proteomic analysis. A protein standard marker is labelled on the left side of the gel, while the approximate size range of each fraction is labeled to the right (both in kDa). Download FIG S1, PDF file, 0.8 MB. Copyright © 2021 Gimza et al. 2021 Gimza et al. https://creativecommons.org/licenses/by/4.0/ This content is distributed under the terms of the Creative Commons Attribution 4.0 International license.

10.1128/mBio.03288-20.2 FIG S2 Hypervirulence is not observed for *aur* or *scpA* single mutants. The wild-type (WT) and protease mutants noted above were separately inoculated via tail vein injections into groups of 10 CD-1 mice, at 1 × 10^8^ cells. Infections were allowed to progress for 6 days or until mice reached a premoribund state (measure of mortality). Statistical significance was determined used a log rank test (****, *P* < 0.0001 [relative to the wild-type strain]). WT and protease-null mutant, *n* = 120 mice per strain. *aur* and *scpA*, *n* = 10 mice per strain. Download FIG S2, PDF file, 0.1 MB. Copyright © 2021 Gimza et al. 2021 Gimza et al. https://creativecommons.org/licenses/by/4.0/ This content is distributed under the terms of the Creative Commons Attribution 4.0 International license.

10.1128/mBio.03288-20.3 FIG S3 An *aur scpA* double mutant still possesses secreted protease activity. To assess proteolytic activity, zymography was performed using 15-h culture supernatants isolated from wild-type, protease-null mutant, and *aur scpA* mutant strains. All strains were standardized to each other based on the optical density before concentrating culture supernatants. Download FIG S3, PDF file, 0.8 MB. Copyright © 2021 Gimza et al. 2021 Gimza et al. https://creativecommons.org/licenses/by/4.0/ This content is distributed under the terms of the Creative Commons Attribution 4.0 International license.

### Zymography.

The wild-type, protease-null mutant, and *aur scpA* mutant strains were grown in biological triplicates as described above, and samples were taken at 15 h. To assess the proteolytic activity, zymograms were performed using concentrated supernatants, as described by us previously (48).
