# Supplementary material for: Unraveling the Impact of Secreted Proteases on Hypervirulence in Staphylococcus aureus
Source: mBio. 2021 Feb 23;12(1):e03288-20. doi: 10.1128/mBio.03288-20 (PMC8545110; doi:10.1128/mBio.03288-20)
Supplement: TABLE S1 [file mbio.03288-20-st001.pdf]

Table S1. Primers used in this study.

Primer	Sequence[a]	Description
OL4212	ATGgaattcGTAACTAACTTGAGTACACGTG	*aur* KO A frag
OL4213	ATGacgcgtGTATGAAGCATGGAATGAAGTAG	*aur* KO A frag
OL4214	ATGacgcgtTGCCATACTTGTAAATGCATATC	*aur* KO B frag
OL4215	ATGggtaccGATTGGAGTAACAGTTGTTGAA	*aur* KO B frag
OL4220	ATGacgcgtGATAAACCCAGCGAACCATTTG	Kanamycin cassette
OL4221	ATGacgcgtATCGATACAAATTCCTCGTAGGC	Kanamycin cassette
OL4216	ATGggtaccTTTAGAGAAAGTTGAAGATGAAACTGTG	*sspB* KO A frag
OL4217	ATGagatctGCCTATTACATTTATCATTCAATCG	*sspB* KO A frag
OL4218	ATGagatctGCATTGAAACCATTATGATGCT	*sspB* KO B frag
OL4219	ATGggtaccTATTCGTTGCAACTTTGACAAC	*sspB* KO B frag
OL4473	ATGgagctcCTAGCTTAGCCTGCCATGATG	pKan inverse
OL4474	ATGgagctcGGTCAATCGAGAATATCGTCAACTG	pKan inverse
OL4563	ATGgagctcCCAGTCATACCAATAACTTAAGGG	Chloramphenicol cassette
OL4564	ATGgagctcCTTATCAATTTGTTGCAACGAACAG	Chloramphenicol cassette
OL4618	AGGAGCGATTTACATATGAGTTATG	pCM screening
OL4619	GAGATACCCAGATCATATGAAACAG	pCM screening
OL535	ATGATGgcatgcGGTGTGTAGACATCTTCACCC	*aur* screening
OL3402	TCAAGATTACGCTGTTACTGATGT	*aur* screening
OL15	ATCccgcggGTGTTACCCCTATTGCAAACGC	*scpA* screening
OL16	GCTggatccCATCGTATAGCCTGCACACCAACC	*scpA* screening
OL3496	CGGTAATAGAGGGAAACGATGAC	Tn::Tet screening
OL3528	TTCCGGTGTAGTTGTAGGTAAAG	*sspA* screening
OL3529	TGGTTCATCTGGGTTGTTAGG	*sspA* screening
OL147	GCCAAAGCCGATTCACACTC	*sspB* screening
OL148	CCTGCACACCATGAGTTATCG	*sspB* screening
OL1979	ATGATGggatccGTTGGATGTGAAAGATTG	*spl* screening
OL3872	TCTTCATCTGCCCATGCTTC	*spl* screening
OL5135	CAAATGAAGTAGCTGGTAATAC	*lukA* screening
OL5136	ATTCACATGTCGATACATATCAAC	*lukA* screening
OL5679	CAGTGTTATGATGGAGTTACAAT	*spn screening*
OL5680	GTGAGTTAATCACTGACTTCTA	*spn screening*
OL5315	CGTCTCCATTTGGCAGTGA	*sek* screening
OL5316	GAACTGGTCAAGCAGAGAG	*sek* screening
OL5125	ATGgaattcACGAAAATATCATTTCTGTCCCACTC	SAUSA300_0964 KO A frag
OL5126	ATGagatctAATGTAACCTCCGAATGATAAGTCA	SAUSA300_0964 KO A frag
OL5127	ATGagatctCCCTCATCACTATACTGCAAATAAAATG	SAUSA300_0964 KO B frag
OL5128	ATGggtaccGACCTCCTAATACTACAAATGTAGGGC	SAUSA300_0964 KO B frag

[a]Restriction sites are underlined lowercase letters
