# Supplementary material for: Unraveling the Impact of Secreted Proteases on Hypervirulence in Staphylococcus aureus
Source: mBio. 2021 Feb 23;12(1):e03288-20. doi: 10.1128/mBio.03288-20 (PMC8545110; doi:10.1128/mBio.03288-20)
Supplement: TABLE S2 [file mbio.03288-20-st002.pdf]

Table S2: Secreted proteins with increased abundance in both the *aur*/*scpA* mutant and protease-null mutant strain, but not in the wild-type.

Accession Number	Gene	Description	Fold Change *aur*/*scpA* vs WT	Fold Change Protease-Null vs WT
SAUSA300_0964		Chitinase-related protein	53.3	66.4
SAUSA300_0277	*essH*	Peptidoglycan Hydrolase	30.9	47.2
SAUSA300_0409	*spin*	Staphylococcal peroxidase inhibitor	30.0	38.1
SAUSA300_1052	*ecb*	Complement inhibitory protein	25.2	24.5
SAUSA300_2364	*sbi*	Immunoglobulin-binding protein	14.5	15.5
SAUSA300_0146		Hypothetical protein	14.3	15.4
SAUSA300_1975	*lukA*	Leukotoxin	11.9	4.1
SAUSA300_2561	*phoB*	Alkaline phosphatase	5.1	6.3
SAUSA300_2579	*lytZ*	Putative peptidoglycan hydrolase	1.7	6.0
SAUSA300_0424.1	*psmα4*	Phenol-soluble modulin alpha 4	3.6	2.3
SAUSA300_1988	*hld*	Delta-hemolysin	2.7	2.6
SAUSA300_1974	*lukB*	Leukotoxin	3.4	2.4
SAUSA300_0800	*sek*	Enterotoxin K	2.1	2.5
SAUSA300_0602		Hypothetical protein	1.9	2.9
SAUSA300_0955	*atl*	Autolysin	2.0	2.5
