# Supplementary material for: Unraveling the Impact of Secreted Proteases on Hypervirulence in Staphylococcus aureus
Source: mBio. 2021 Feb 23;12(1):e03288-20. doi: 10.1128/mBio.03288-20 (PMC8545110; doi:10.1128/mBio.03288-20)
Supplement: FIG S1 [file mbio.03288-20-sf001.pdf]

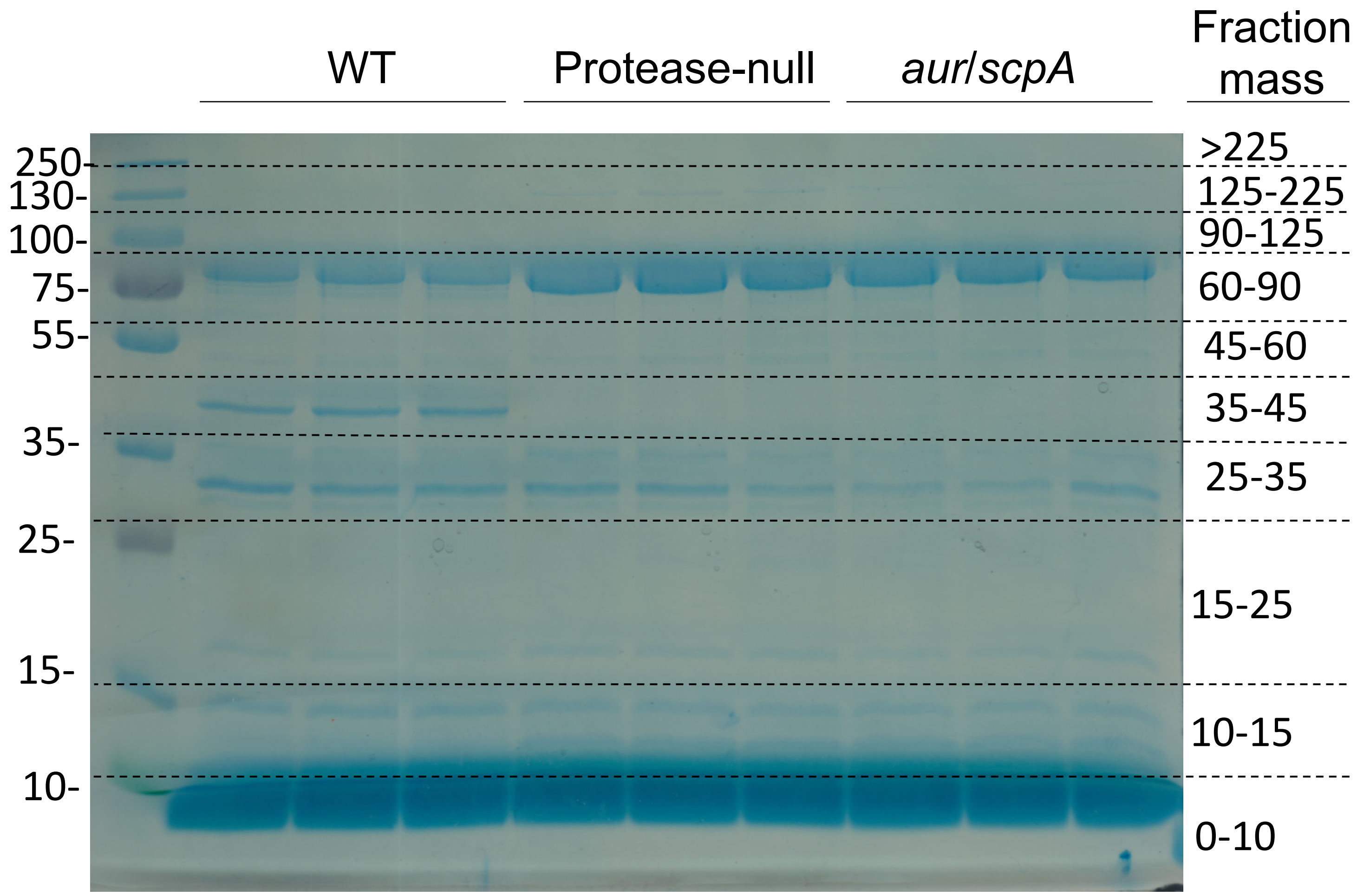

Supplemental Figure S1. Depiction of SDS-PAGE gel sectioning by mass fraction for proteomic analysis. A protein standard marker is labelled on the left side of the gel, whilst the approximate size range of each fraction is labelled to the right (both in kDa).
