# Supplementary material for: Unraveling the Impact of Secreted Proteases on Hypervirulence in Staphylococcus aureus
Source: mBio. 2021 Feb 23;12(1):e03288-20. doi: 10.1128/mBio.03288-20 (PMC8545110; doi:10.1128/mBio.03288-20)
Supplement: FIG S2 [file mbio.03288-20-sf002.pdf]

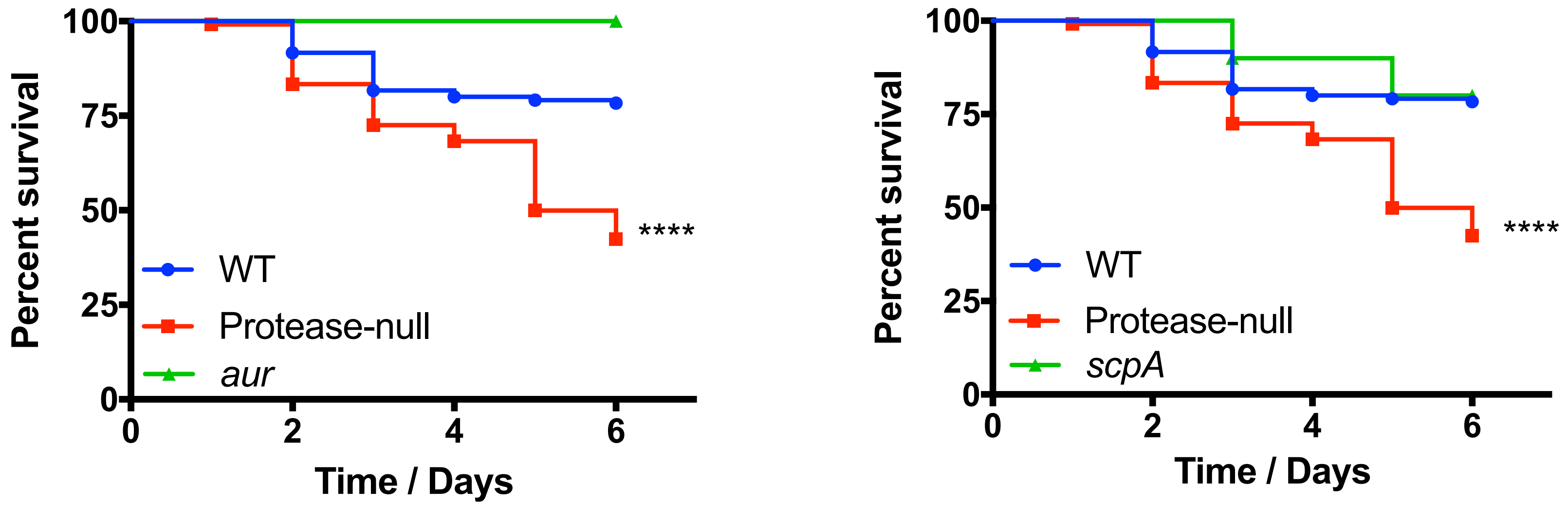

Supplemental Figure S2. Hypervirulence is not observed for *aur* or *scpA* single mutants. The wild-type (WT) and protease mutants noted above were separately inoculated via tail vein injections into groups of 10 CD-1 mice, at 1 x 10^8 cells. Infections were allowed to progress for 6 days or until mice reached a premoribund state (measure of mortality). Statistical significance was determined used a Logrank test (****, $P < 0.0001$; relative to the wild-type strain). WT and protease-null mutant, n = 120 mice per strain. *aur* and *scpA*, n = 10 mice per strain.
