# Supplementary material for: Unraveling the Impact of Secreted Proteases on Hypervirulence in Staphylococcus aureus
Source: mBio. 2021 Feb 23;12(1):e03288-20. doi: 10.1128/mBio.03288-20 (PMC8545110; doi:10.1128/mBio.03288-20)
Supplement: FIG S3 [file mbio.03288-20-sf003.pdf]

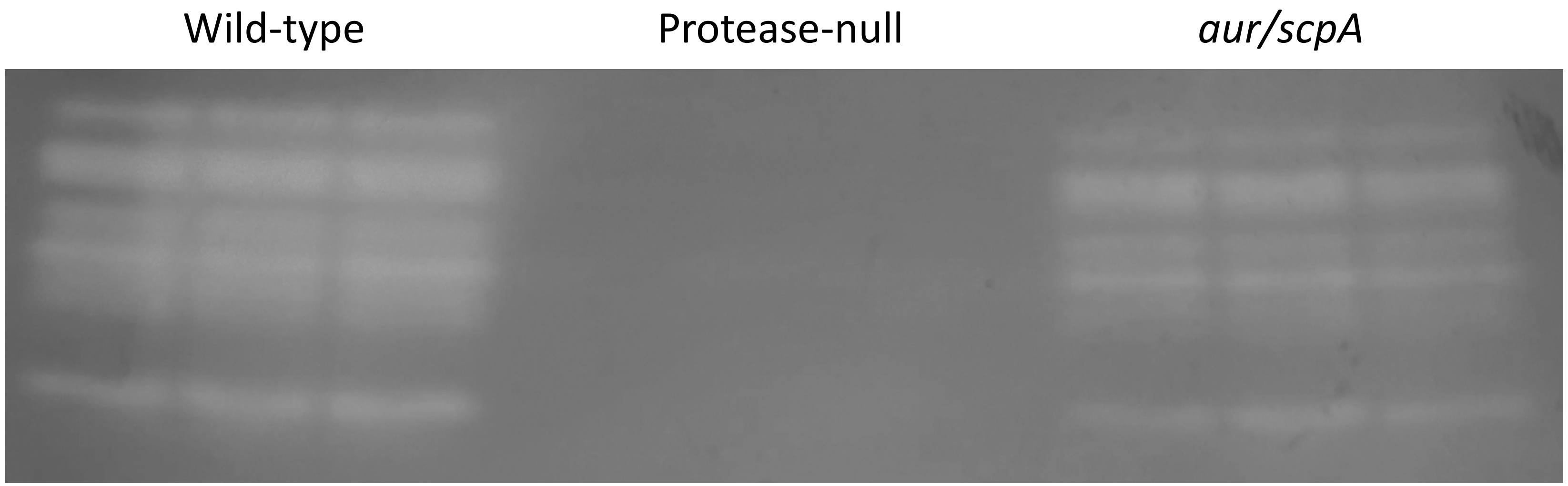

Supplemental Figure S3. An *aur/scpA* double mutant still possesses secreted protease activity. To assess proteolytic activity, zymography was performed using 15 hr culture supernatants isolated from the wild-type, protease-null, and *aur/scpA* mutants. All strains were standardized to each other by optical density before concentrating culture supernatants.
